# Prevalence and prognostic value of baseline sarcopenia in hematologic malignancies: a systematic review

**DOI:** 10.3389/fonc.2023.1308544

**Published:** 2023-12-14

**Authors:** Xiaofeng Zeng, Liying Zhang, Yu Zhang, Shuli Jia, Taiping Lin, Xuman Zhao, Xiaoli Huang

**Affiliations:** ^1^ The Center of Gerontology and Geriatrics, National Clinical Research Center for Geriatrics, West China Hospital, Sichuan University, Chengdu, China; ^2^ Sichuan University Library, Sichuan University, Chengdu, China

**Keywords:** hematologic malignancies, sarcopenia, prevalence, prognosis, systematic review

## Abstract

**Background:**

The correlation between sarcopenia and hematological malignancy prognosis is still controversial. Design: A systematic review and meta-analysis. Objectives: To explore sarcopenia’s prevalence and prognostic value in hematologic malignancies.

**Data sources and methods:**

We searched Embase, MEDLINE, and Cochrane Library through Ovid SP using an appropriate search strategy on August 28, 2022, and updated the search results on January 9, 2023. Study quality was assessed using the Newcastle-Ottawa scale. The pooled prevalence of sarcopenia was calculated with a 95% confidence interval (CI). Relationships between sarcopenia and prognostic value were expressed as hazard ratio (HR) and 95% CI. HR means the probability of something undesirable, i.e., death or disease progression.

**Results:**

The search identified more than 3992 studies, and 21 (3354 patients, median or mean age ranging from 36 to 78 years) were finally included. The risk of bias in the studies was low to medium. All included studies were diagnosed based on low muscle mass (LMM). Muscle mass was assessed mainly through imaging technologies, and different cut-offs were applied to determine LMM. The prevalence of sarcopenia was 44.5%, which could fluctuate by age. Subgroup analysis showed that older people had a higher sarcopenic rate than the non-elderly group. Sarcopenia resulted in an inferior prognosis [overall survival: HR 1.821, 95% CI 1.415-2.343; progression-free survival: HR 1.703, 95% CI 1.128-2.571).

**Conclusion:**

Sarcopenia has a prevalence of over 30% in malignant hematologic patients and is associated with a poorer prognosis. Future studies with a standardized sarcopenia diagnostic criterion were needed to investigate sarcopenia’s prevalence and prognostic effects in hematologic malignancies.

## Introduction

With the aging of the population, patients with hematological malignancies have attracted attention. Acute myeloid leukemia (AML) incidence and prognosis are directly related to age; elderly patients account for approximately 70% (1) of patients. Diffuse large B-cell lymphoma (DBLCL) has a median age of 70 at diagnosis (2). In Hodgkin lymphoma (HL), approximately 25% of patients are over 60 years old at diagnosis (3). The median diagnostic age of multiple myeloma (MM) is approximately 70 years (4).

When it comes to the treatment of hematologic malignancies, chemotherapy has the highest status. Other therapies include radiotherapy, immunotherapy, targeted therapy, and hematopoietic stem cell transport (HSCT). HSCT, usually applied when patients arrive at complete remission after chemotherapy, is seen as the only way to cure completely high-risk patients of hematologic malignancies (5–8). Identifying patients who can tolerate intensive induction chemotherapy and HSCT treatment is essential (9). However, the most used prognostic tools for hematologic malignancies only consist of some clinical characteristics (age, disease stage, ECOG performance status, serum lactate dehydrogenase level, etc.) validated over two decades ago (10). In clinical cases, assessing a patient’s clinical situation is primarily based on physician-subjective assessment, resulting in increased inter-observer differences and reduced accuracy in predicting survival (11, 12). To identify patients with an aggressive disease course, developing prognostic and predictive markers is imperative.

Sarcopenia is a skeletal muscle failure defined as a loss of lean muscle strength and mass, with or without impaired muscle function (13). It is a disease that is more likely to appear in older people while not elderly-specially. Sarcopenia has been associated with worse prognosis and increased treatment toxicities in neoplastic patients (14), such as esophagogastric, colorectal, breast, lung, liver, and renal cell cancer (15–23). Studies about sarcopenia’s predictive value in hematologic malignancies are increasing (9, 24–30). However, the findings present significant disagreement. These studies reported a wide range in sarcopenia prevalence.

The correlation between sarcopenia and the prognosis of hematological malignancies remains controversial. Some studies have shown that sarcopenia is a poor prognostic factor for patients with hematological malignancies (24). Some studies have suggested that sarcopenia is a poor prognostic factor for male patients with hematological malignancies (27). Some studies have indicated no correlation between sarcopenia and the prognosis of patients with hematological malignancies (30).

Alexey Surov and Andreas Wienke performed a meta-analysis to disclose the prognostic influence of sarcopenia in hematologic malignancies (31). We reviewed this meta-analysis and found that the authors included articles with variable timing of sarcopenia assessment, with some reports assessing sarcopenia before any treatment and others assessing sarcopenia before HSCT. Meanwhile, the inclusion of few studies makes the conclusion unconvincing and difficult to apply widely. As a result, it is meaningful to conduct an overall systematic review and meta-analysis to investigate the sarcopenic prevalence and prognostic value of hematologic malignancies and provide guidance on the treatment options available to patients with hematology malignancies.

## Methods

We performed this systematic review according to the Preferred Reporting Items of Systematic Reviews and Meta-Analyses (PRISMA) guidelines (32). The review was not registered.

### Eligibility and exclusion criteria

Inclusion criteria: (i) research participants must be adult patients with hematological malignancies, without a second active malignant tumor or a history of a hematologic malignant tumor in the past; (ii) sarcopenia or less skeletal muscle mass (LSMM) was assessed before any treatment (for this analysis, we only correlated baseline results of sarcopenia with clinical outcomes); (iii) prognostic effects of sarcopenia, e.g., overall survival (OS) or progression-free survival (PFS) were analyzed in all the included patients; (iv) observational studies; (v) hazard ratios (HR) and their respective 95% confidence intervals (CIs) as a measure of effect estimators (HR refers to the probability of something undesirable happening, i.e., death or disease progression); (vi) published in English. Exclusion criteria: (i) no use of a standard or convinced method to diagnose sarcopenia; (ii) no use of a proper sarcopenia or LSMM cut-off value; (iii) no report of any prognostic outcomes; (iv) reviews, case reports, conference abstracts, letters, comments, or other types of publications that did not report complete data.

### Outcomes

(1) Sarcopenic prevalence in patients with hematologic malignancies (2). Prognostic values. OS is from diagnosis to death for any reason or last follow-up. PFS is from diagnosis to the first disease progression, relapse, and death for any cause or last follow-up.

### Search strategy

We implemented a thorough literature search in MEDLINE, EMBASE, and Cochrane Library using Ovid SP on August 28, 2022. We used an appropriate search strategy designed by a professional librarian (YZ). The detailed messages of the search strategy are depicted in **Table S1**. Additionally, references from the selected literature were screened for potentially included studies. Moreover, we updated the search results on January 9, 2023.

### Study selection

Two reviewers (XFZ and YZ) independently assessed the titles and abstracts of all publications to confirm possible relevant studies. Then, full-text censoring was conducted when either reviewer considered the article in need of further exploration. An additional rater was consulted in the case of discrepancies (XLH); if two or more studies used data from the same cohort, the largest sample size was included in the analysis.

### Data extraction

Two reviewers (XFZ and LYZ) independently extracted data using a well-designed form, which includes the following variables: the name of the first author, publication year, country, study design, subjects enrolled interval, sample size, male proportion, subjects’ age, disease type, treatment, chemotherapy cycles, follow-up duration, sarcopenia diagnostic criteria, the prevalence of sarcopenia, and the hazard ratio (HR) and 95% confidence interval (CI) for disease outcomes like OS or PFS. An additional rater was consulted in the case of discrepancies (XLH).

### Quality assessment

Two reviewers (XFZ and LYZ) independently evaluated the quality of the retrospective cohort research using the Newcastle-Ottawa Scale (NOS) (33). Disagreement was resolved by the third reviewer (XLH). The NOS ranges from 0-9 points, with ≥7 points seen as high quality, 4-6 points as moderate quality, and <4 as low quality.

### Data analysis

We used STATA/MP (Version 14.0, StataCorp, College Station, TX, USA) software to perform the meta-analysis. Heterogeneity was estimated by the I^2^ test, with I^2^ values greater than 25%, 50%, and 75%, respectively, representing low, moderate, and high heterogeneity (34). The fixed-effects model was employed to calculate the pooled sarcopenia prevalence with a 95% CI when the I^2^ index implied a low heterogeneity; otherwise, the random-effects model was applied. To confirm the effect of sarcopenia on the disease results, like OS and PFS, the HR and 95% CI were retrieved and used for meta-analysis. Data from multivariate analyses were retrieved for meta-analysis when we could extract HR and 95% CI from univariate and multivariate analyses. To investigate possible reasons for heterogeneity, we performed subgroup analyses and meta-regression.

### Sensitivity analysis and publication bias

We conducted sensitivity analysis by omitting single studies from pooled analyses. Egger’s test (35) and the Begg test (36) assessed publication bias (P < 0.05).

## Result

### Study selection

**Figure 1:**
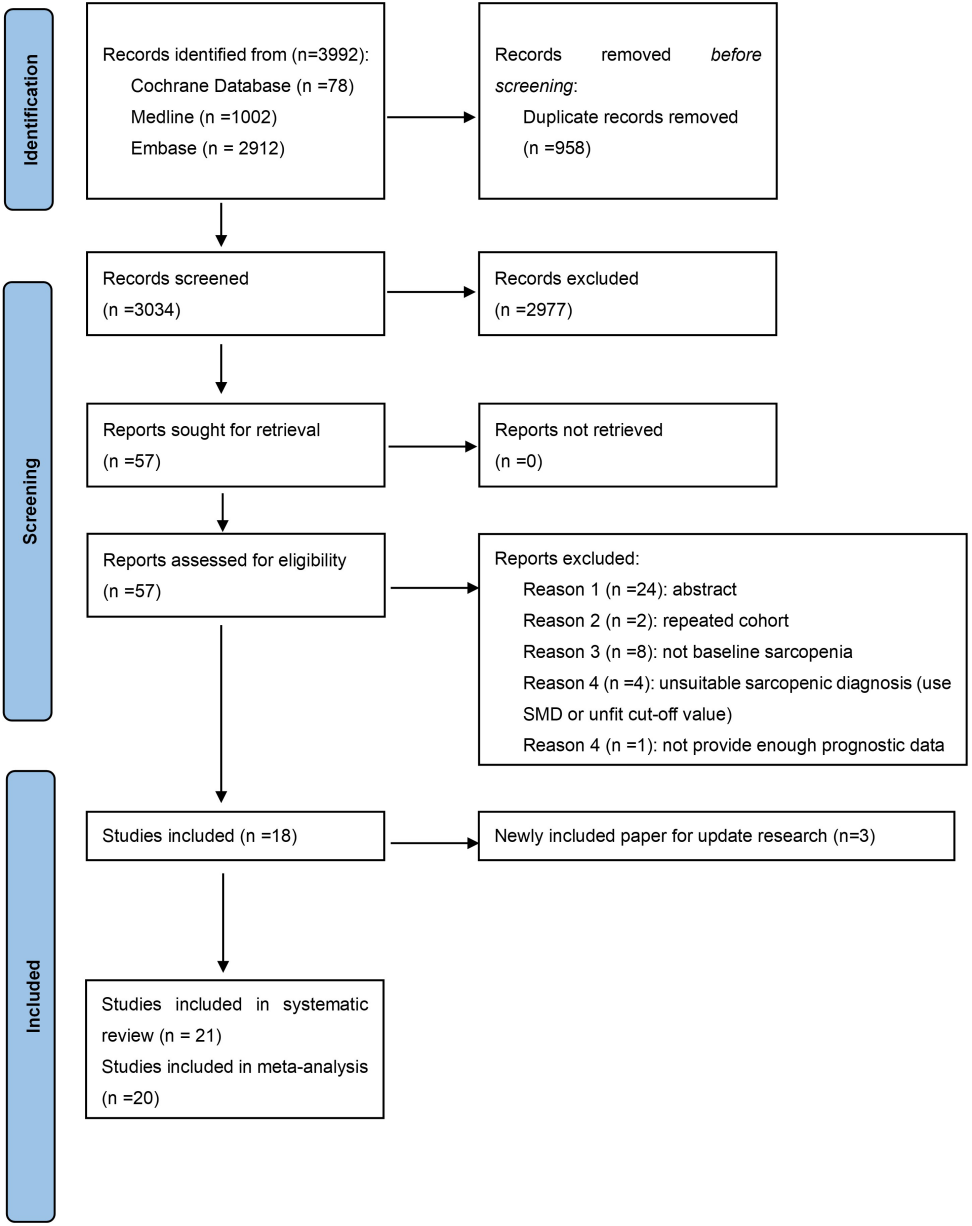
The flow chart of the literature selection. SMD, Skeletal muscle density.

In the first round of study detection, we found 3992 studies, of which 958 were duplications. After the review of titles and abstracts, 2977 studies were excluded due to not meeting the research topic. A total of 57 studies underwent full-text checking, and 18 of these papers were included. The exact reasons for excluding articles in full-text checking are displayed in **Table S2**. No extra paper was identified from the manual reference review of the included articles. Then, during the updated search, we found 3 recently published studies compliant with the inclusion criteria. As a result, 21 studies are included in the systematic review and 20 in the meta-analysis (1 did not provide relevant data). The process of study selection is exhibited in **Figure 1**.

### Study characteristics

The characteristics of the 21 included studies are summarized in **Table 1**. The sample sizes ranged from 43 to 656, with 3354 total patients and a median (or mean) age ranging from 36 to 78 years. All of these studies were retrospective cohorts published after 2013. In total, 3 studies were conducted in AML populations (9, 24, 37), 15 were conducted in lymphoma populations (DLBCL occupied for > 90%) (25–28, 30, 38–47), and 3 were conducted in MM populations (29, 48, 49). The participants came from various regions: 12 studies were conducted in Europe, 6 in Asia, and 3 in the USA. All papers except one provided treatment messages, and most patients received chemotherapy. Notably, we can see that most DLBCL patients received the classic R-CHOP regimen.

**Table 1:** The characteristics of the included studies.

Authors, year	Country	Enrolled interval	Study size (Male%)	Population age (years)	Disease type	Sarcopenia diagnostic criteria	Sarcopenia prevalence	Elderly sarcopenia prevalence
Nandakumar, 2023	USA	01/2005-07/2019	322 (62%)	median 66 (range 37–95)	MM	LMM (CT)	53.1%	–
Ferraro, 2022	Germany	2013−2019	72 (51.4%)	median 68 (range 23−81)	PCNSL	LMM (CT)	51.4%	–
Albano, 2022	Italy	01/2010-06/2021	88 (47%)	mean 72.8 (range 65–91)	HL	LMM (PETCT)	65.9%	
Sun, 2022	China	02/2012-08/2021	227 (48.0%)	median 64 (range 24-87)	AML	LMM (BIA)	18.1%	>60 years old, 21.0% (32/152)
Albano, 2022	Italy	01/2010-12/2020	53 (74.0%)	mean 72.7 (SD 5.6, range 66–88)	Mantle Cell Lymphoma	LMM (PETCT)	60.4%	
Lucijanić, 2021	Croatia	11/2003-12/2018	49 (51.0%)	median 36	cHL	LMM (CT)	NA	–
Zilioli,2021	Italy	01/2006-12/2018	154 ^a^ (50.6%)	median 71 (range > 64)	cHL	LMM (CT/PETCT)	35.5% in males	
Leone,2021	Italy	01/2010-01/2020	43 (34.9%)	mean 61 (SD 10)	PCNSL	LMM (CT)	30.2%/25.6%	–
Koyuncu,2021	Turkey	2015-2020	111 (48.7%)	median 64 (range 37–84)	MM	LMM (PETCT)	41.4%	–
Jung, 2021	Korea	2012-2017	96 (52.1%)	median 58 (range 18–84)	AML	LMM (CT)	37.5%	–
Jullien, 2021	France	2013-2015	656 (55.9%)	median 48 (IQR 38–55)	DLBCL	LMM (PETCT)	34.3%	–
Furtner,2021	Austria	2005-2018	128 (51.6%)	mean 62.7 (range 23–84)	PCNSL	LMM (MRI)	35.9%	–
Besutti, 2021	Italy	01/2014-12/2017	116 (51.7%)	mean 63.7 (SD 16.4)	DLBCL	LMM (PETCT)	25%	–
Iltar, 2020	Turkey	03/2013-12/2019	120 (55.0%)	mean 59.11 (SD 13.12, range 52–68)	DLBCL	LMM (CT)	54.2%	–
Nakamura, 2019	Japan	12/2004-10/2016	90 (56.7%)	median 59 (range 18–84)	AML	LMM (CT)	43.3%	(≥ 60 years old), 40.9% (18/44)
Burkart, 2019	USA	2000-2015	109 ^b^ (48.6%)	median 64 (sarcopenia 59.8 ± 15; non-sarcopenia 58.5 ± 15.7)	aggressive B-NHL^c^	LMM (PETCT)	67.9% in males	–
Xiao, 2016	USA	10/1998-10/2008	522 (97.7%)	mean 64.4 (SD 11.5)	DLBCL	LMM (CT)	46.9%	–
Takeoka, 2016	Japan	05/2009-01/2015	56 (33.9%)	median 71 (range 65-75)	MM	LMM (CT/PETCT)	66.10%	–
Go, 2016	Korea	06/2003-02/2015	187 (59.9%)	sarcopenia: median 66.5 (range 24–89); non-sarcopenia: median 60 (range 17–86)	DLBCL	LMM (CT)	24.6%	–
Nakamura, 2015	Japan	06/2004-05/2014	207 (58.5%)	median 67 (range 19–86)	DLBCL	LMM (CT)	55.6%	–
Lanic, 2014	France	11/2005-01/2011	82 (43.9%)	mean 78 (range 70-95)	DLBCL	LMM (CT)	54.9%	

MM, multiple myeloma; PCNSL, primary central nervous system lymphoma; HL, Hodgkin lymphoma; AML, acute myeloid leukemia; DBLCL, diffuse large B-cell lymphoma. ^a^76 males were included in the meta-analysis. ^b^53 males were included in the meta-analysis. ^c^Include diffuse large B-cell lymphoma (DLBCL), mantle cell lymphoma (MCL) and Burkitt lymphoma.

### Risk of bias

The NOS grades of the included papers are shown in **Table S3**. The included studies had moderate to high quality, with the NOS scores ranging from 5 to 8.

### Diagnostic method and prevalence of sarcopenia

Regarding the definition of sarcopenia, all included studies were diagnosed based on low muscle mass (LMM). In addition to one study that used Bioelectrical impedance analysis (BIA) (24), the rest of the 20 studies applied imaging technologies [Computed Tomography (CT), Positron Emission Tomography/CT (PET/CT), or Magnetic Resonance Imaging (MRI)] to measure skeletal muscle mass (SMM): 15 studies measured skeletal muscle mass index (SMI) (13 studies evaluated on L3 level, 1 study on L1 level, and 1 study on T4 level); 3 studies measured psoas muscle index (PMI); 2 studies measured temporal muscle thickness (TMT). As for diagnostic criteria (cut-off values), 11 studies were chosen from former research, 7 studies were identified through the ROC curve or survival curve, and the remaining 3 studies were defined as 20% quantile, lower quartile, and median, respectively (**Table 2**).

**Table 2:** Skeletal muscle mass measurement approaches and cutoff thresholds.

Authors, year	Skeletal muscle mass assessment	Sarcopenia diagnosis	Cut-off value definition
Nandakumar, 2023	CT	SMI-L3	from a former study: 55 cm^2^/m^2^ for male and 39 cm^2^/m^2^ for female
Ferraro, 2022	CT	SMI-L3	from a former study: <52.4 cm^2^/m^2^ for males and <38.5 cm^2^/m^2^ for females
Albano, 2022	PETCT	SMI-L3	from a former study: 55 cm^2^/m^2^ for male and 39 cm^2^/m^2^ for female
Sun, 2022	BIA	SMI	according to EWGSOP2: ASM <20 kg (for male) or <15 kg (for female), and/or SMI <7.0 kg/m^2^ (for male) or <5.5 kg/m^2^ (for female)
Albano, 2022	PETCT	SMI-L3	ROC curve: 53 cm^2^/m^2^ for male, 45.6 cm^2^/m^2^ for female
Lucijanić, 2021	CT	PMI-L3	ROC curve: <582mm^2^/m^2^
Zilioli,2021	CT or PETCT	SMI-L3	ROC curve: 45 cm^2^/m^2^ for male patients
Leone,2021	CT	TMT	−2.5 SD from the mean L3-SMI value of a healthy young reference population: 41.4 cm^2^/m^2^ in male and 31.0 cm^2^/m^2^ in female; cut-off value for TMT: 6.3 mm in male and 5.2 mm in female
Koyuncu,2021	PETCT	PMI-L3	from a Turkish population study: PMI <540 mm²/m²for male and <360 mm²/m²for female
Jung, 2021	CT or PETCT	SMI-L1	the cutoff finder method was used to determine the appropriate SMI cutoff values of where the difference in survival curves was maximized: 40.79 cm^2^/m^2^ for male, 31.6 cm^2^/m^2^ for female
Jullien, 2021	PETCT	SMI-L3	from a former study: 55 cm^2^/m^2^ for male and 39 cm^2^/m^2^ for female
Furtner,2021	MRI	TMT	from a former study: male ≤6.3 mm; female ≤5.2 mm
Besutti, 2021	PETCT	SMI-L3	from a former study: <43 cm^2^/m^2^ for male with BMI <25, <53 cm^2^/m^2^ for male with BMI ≥25, and <41 cm^2^/m^2^ for female
Iltar, 2020	CT	PMI-L3	ROC analysis: ≤440.4 mm^2^/m^2^ in males and ≤306.87 mm^2^/m^2^ in females
Nakamura, 2019	CT	SMI-L3	ROC analysis: < 48.4 cm^2^/m^2^ in males and < 33.5 cm^2^/m^2^ in females
Burkart, 2019	PETCT	SMI-L3	SMI below the median muscle mass measured in the study population: SMI <56.8 cm^2^/m^2^ in male and < 47.4 cm^2^/m^2^ in female
Xiao, 2016	CT	SMI-L3	from a former study: SMI <53 cm^2^/m^2^ in male and <41 cm^2^/m^2^ in female
Takeoka, 2016	PETCT	SMI-L3	from a former study: <43 cm^2^/m^2^ for male with BMI <25, <53 cm^2^/m^2^ for male with BMI ≥25, and <41 cm^2^/m^2^ for female
Go, 2016	CT	SMI-T4*	the lowest sex-specific quartile of the SMI: 440 cm^2^/m^2^ in male and 310 cm^2^/m^2^ in female
Nakamura, 2015	CT	SMI-L3	ROC curve: <47.1 cm^2^/m^2^ in males and <34.4 cm^2^/m^2^ in females
Lanic, 2014	CT	SMI-L3	lie within the inner 80% of the LSMI distribution: < 55.8 cm^2^/m^2^ for male and 38.9 cm^2^/m^2^ for female

SMI=SMA/height², skeletal muscle area (SMA) was assessed from a single axial slice at the level of the third lumbar vertebra (L3) considering psoas, paraspinal, abdominal transverse rectum, internal, and external obliques muscles. PMI = (RPA + LPA)/height², RPA: right psoas muscle area, LPA: left psoas muscle area. TMT: temporal muscle thickness. * Including the pectoralis major and the pectoralis minor.

**Figure 2:**
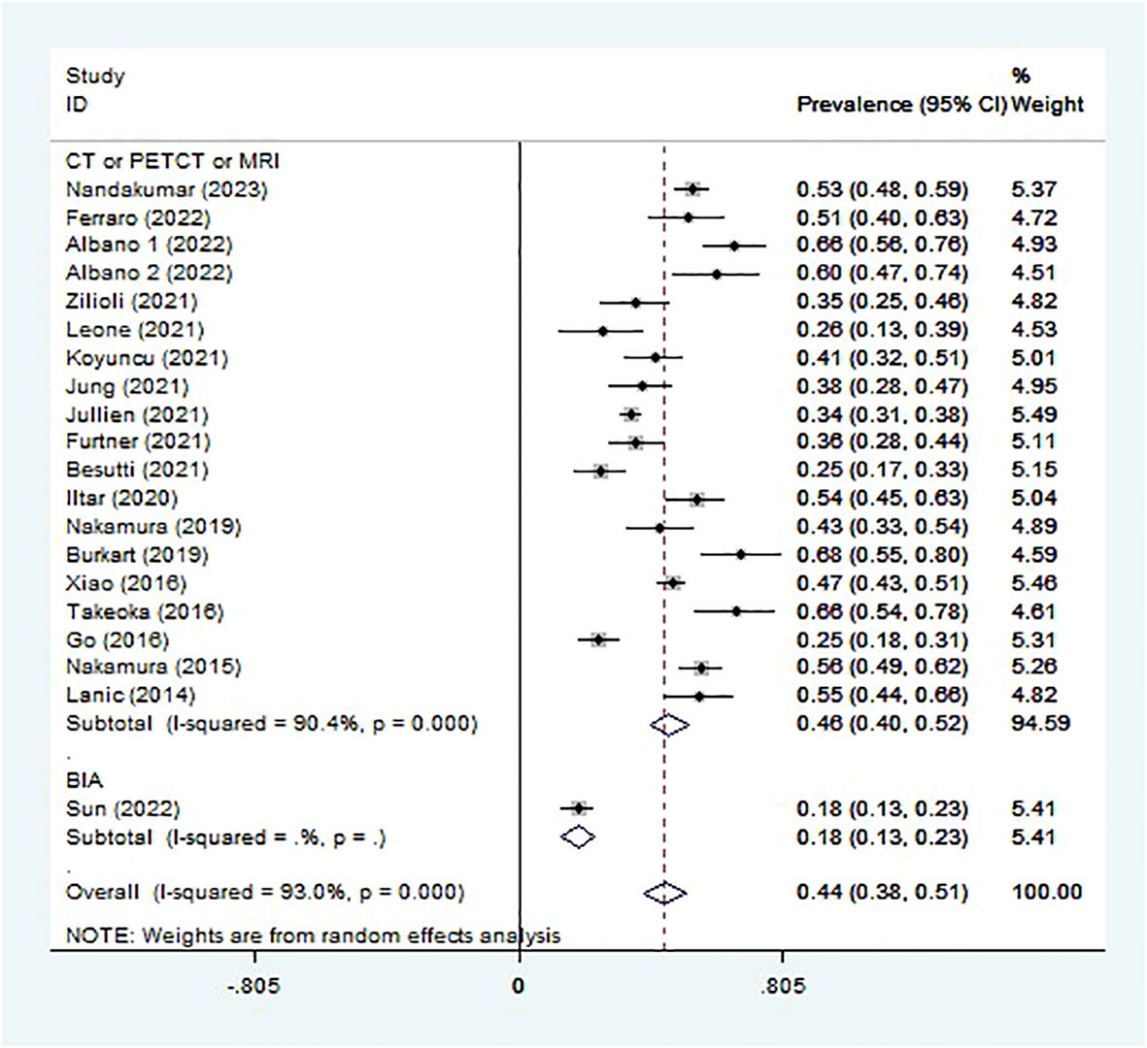
Pooled prevalence of sarcopenia.

Sarcopenia prevalence ranged from 18.1% to 67.9% (**Table 1**), and the pooled prevalence was 44.5% (95% CI 38.1-50.9%, I^2 = ^93.0%; **Figure 2**). Lucijani´c et al. (25) did not report a sarcopenia prevalence or low PMI rate, so this report is not included in the meta-analysis. The random-effects model was selected.

### Meta−regression of prevalence

Median or mean age affects the prevalence of sarcopenia (regression coefficient 0.011, 95% CI 0.001 to 0.021, p = 0.027, 20 studies, 3305 patients) (**Figure S1**). Furthermore, we did a meta-regression to investigate the influence of different regions, while no effect was displayed (**Figure S2**).

### Subgroup analysis: age, sarcopenia diagnosis, and disease type

We divided the enrolled studies into an elderly group and a non-elderly group with a mean (median) age of 65. Older people had a higher sarcopenic rate (55.1%,95% CI 49.2-61.0%, 8 studies, 956 patients) than the non-elderly group (37.5%, 95% CI 30.4-44.6%, 12 studies, 2349 patients) (**Figure S3**).

Subgroup analysis was conducted in different ways to diagnose sarcopenia. The sarcopenia prevalence in studies assessed by imaging technologies (CT or PET/CT or MRI) (45.9%, 95% CI 40.2-51.7%, 19 studies, 3078 patients) was higher than that assessed by BIA (18.1%, 95% CI 13.1-23.1%, 1 study, 227 patients) (**Figure 2**). The sarcopenia prevalence varied for different scan sites used to assess muscle mass. The prevalence of sarcopenia was highest in PMI estimated at the L3 level (47.8%, 95% CI 35.3-60.4%, 2 studies, 231 patients), followed by SMI at the L1, L3, or T4 level (47.6%, 95% CI 40.8-54.4%, 14 studies, 2676 patients), the third was TMT (32.0%, 95% CI 22.2-41.8%, 2 studies, 171 patients) (**Figure S4**).

In addition, sarcopenia prevalence was highest in MM (52.9%, 95% CI 41.5-64.3%, 3 studies, 489 patients), followed by lymphoma (47.2%, 95% CI 38.9-55.4%, 11 studies, 2160 patients), PCNSL (37.8%, 95% CI 24.6-51.0%, 3 studies, 243 patients), and AML (32.5%, 95% CI 15.5-49.5%, 3 studies, 413 patients) (**Figure S5**).

### Impact of sarcopenia on survival outcomes (OS and PFS)

**Figure 3:**
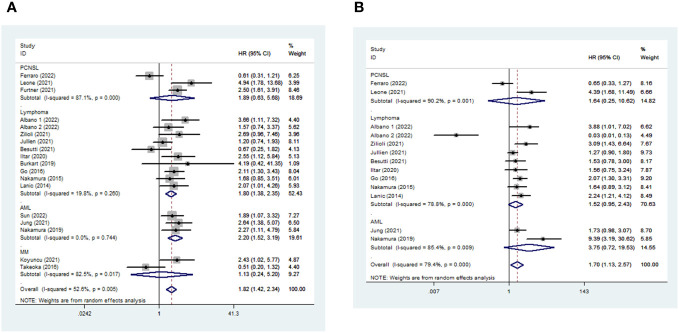
Impact of sarcopenia on OS (a) and PFS (b) in hematologic malignancies. HR means the probability of death (a) or disease progression (b).

The median follow-up duration ranged from 13.8 months to 72 months. In total, 18 and 13 studies were included in the meta-analysis of OS and PFS, respectively (**Table S4**). Sarcopenic patients had a poorer OS than non-sarcopenia [pooled HR 1.821, (95% CI 1.415-2.343), I^2 = ^52.6%, 2461 patients, **Figure 3A**]. Furthermore, patients with LMM also had a higher risk of shorter PFS [pooled HR 1.703, 95% CI (1.128-2.571), I^2 = ^79.4%, 1886 patients, **Figure 3B**] than those with average muscle mass. The random-effects model was chosen.

Among different kinds of hematologic malignancies, sarcopenic patients had a higher risk of mortality than non-sarcopenic patients [AML: pooled OS (HR 2.203, 95% CI 1.524-3.186, I^2 = ^0%, 3 studies, 413 patients) and pooled PFS (HR 3.746, 95% CI 0.718-19.531, I^2 = ^85.4%, 2 studies, 186 patients); Lymphoma: pooled OS (HR 1.797, 95% CI 1.375-2.347, I^2 = ^19.8%, 10 studies, 1638 patients) and pooled PFS (HR 1.522, 95% CI 0.954-2.428, I^2 = ^78.8%, 9 studies, 1585 patients); PCNSL: pooled OS (HR 1.890, 95% CI 0.629-5.683, I^2 = ^87.1%, 3 studies, 243 patients) and PFS (HR 1.636, 95% CI 0.252-10.623, I^2 = ^90.2%, 2 study, 115 patients); MM: pooled OS (HR 1.126, 95% CI 0.244-5.200, I^2 = ^82.5%, 2 studies, 167 patients)] (**Figure 3**).

Noting the considerable heterogeneity of the pooled OS and PFS, we searched for the source of heterogeneity by omitting an included study, one at a time. When we excluded the study by Ferraro et al. (47), the heterogeneity of pooled OS decreased significantly [pooled HR 1.955, 95% CI (1.562-2.447), I^2 = ^36.9%, 2389 patients, **Figure S6**]. When we excluded three of the articles (26, 37, 47), there was an apparent decrease in the heterogeneity of pooled PFS [pooled HR 1.802, 95% CI (1.497-2.170), I^2 = ^29.8%, 1671 patients, **Figure S7**].

### Meta−regression of prognostic value

For the possible reasons for significant heterogeneity, we performed a series of meta-regressions on age (**Figure S8**), region, the published year of study, and the criteria of sarcopenia diagnosis (data not shown). However, none of these factors impacted pooled OS or PFS value.

### Publication bias and sensitivity analyses

There was no evidence of publication bias in the papers that described the prevalence of sarcopenia (19 studies; Begg’s test: P = 0.230; Egger’s test: P = 0.104; **Figure S9**) or the prognostic value (OS: 18 studies; Begg’s test: P = 0.544; Egger’s test: P = 0.848; PFS: 13 studies; Begg’s test: P = 0.428; Egger’s test: P = 0.862; **Figure S10**) in patients with hematologic malignancies. Sensitivity analysis detected that no individual study significantly affected the pooled prevalence of sarcopenia or pooled prognostic value (OS, PFS) (**Figure S11**).

## Discussion

This systematic review is the first article assessing sarcopenia at baseline (before any treatment) and focusing on the impact of sarcopenia on survival outcomes in hematological malignancy patients. This review depicts the wide-ranging prevalence of sarcopenia in hematologic malignancies. The following reasons might explain the highly-varied prevalence of sarcopenia and enormous heterogeneity (1): the small sample sizes of the included studies (half of the studies had fewer than 100 participants) (2); the variability of assessment technologies and cut-offs (3); the different disease types. There is a large proportion of sarcopenia in patients with hematological malignancies, with an average prevalence of more than 30%; thus, attention should be paid to early diagnosis and treatment of sarcopenia. Moreover, elderly and male patients were more likely to be sarcopenic.

On the other hand, sarcopenic patients had poorer OS and PFS than non-sarcopenic patients. We found that sarcopenic patients in AML and lymphoma were associated with a shorter OS with low intra-study heterogeneity. Meanwhile, sarcopenia would be the risk factor for lymphoma patients to decrease PFS. The survival outcomes were not influenced by patients’ age, region, published study time, and sarcopenia diagnosis criteria. The prognostic effect of sarcopenia differed for gender in the lymphoma subgroup. Some studies reported that the predictive impact of sarcopenia only occurred in male (27, 41, 44).

Sarcopenia is an age-related disease that occurs more frequently in older people. In this review, we found that older people had inferior survival outcomes compared to non-elderly sarcopenic patients (37). Aging and co-morbidities could increase the risk of side effects after anti-tumor therapy (26, 27). Elderly patients are often ineligible or hard to treat in standard chemotherapy (24). When making treatment decisions, clinicians should consider their future survival and quality of life (50).

Meanwhile, sarcopenia is associated with poor tolerance to chemotherapy (51). The main reason why sarcopenic hematologic malignancies have a worse prognosis is intolerance to therapy, which includes a lower rate of response to treatment, a higher risk of side effects (febrile neutropenia, severe anemia, or thrombocytopenia), early discontinuation of therapy, and TRM (9, 38, 42, 43). Sarcopenic patients showed a notably higher rate of infections than non-sarcopenic patients (24). Lower muscle mass is reportedly associated with higher chemotherapy toxicities (16, 42, 42), especially when chemotherapy is administered based on body surface area (44, 52). This method only considers height and weight and does not account for the variability in body composition seen among patients, which can result in different pharmacokinetics of chemotherapy (52). Especially in older people with the coexistence of multiple diseases and the use of numerous medications, assessment of sarcopenia could guide treatment planning and dosing (42).

Sarcopenic patients with hematologic malignancies seemed more suitable for choosing reduced-intensity chemotherapy for safety reasons (53). However, intensive chemotherapy makes patients receive better OS than reduced-intensity regimens (24, 44). Decreasing doses or reducing cycles increases the risk of relapse or progression (37). In elderly patients, researchers found that disease progression was the leading cause of death in sarcopenia and non-sarcopenia patients (37). Thus, clinicians should weigh toxicity against efficacy when making treatment decisions. A comprehensive geriatric assessment of this population, including sarcopenia, facilitates a better prognosis prediction. This way, clinicians might give patients and their caregivers the most comprehensive answers to their condition and treatment options. The correct choice between temporary palliative care and further standard treatment is made for maximum benefit (54).

In this review, we investigated the predictive effect of sarcopenia on the disease outcome of patients with hematologic malignancies. The original definition of sarcopenia by the European Working Group on Sarcopenia in Older People (EWGSOP) was based only on detecting low muscle mass (55). EWGSOP updated the description formally in 2020 (EWGSOP2): sarcopenia is probable when low muscle strength is seen and is confirmed by additional documentation of LMM (13). Since most of the data used in the included studies were from before 2020 (**Table 1**), sarcopenia was defined as a sole loss of muscle mass in this review. However, a standard definition of sarcopenia should be applied in future research.

MRI and CT are considered the gold standard for the non-invasive assessment of the amount of muscle (56). The amount of muscle on CT images of a particular lumbar level (L3) correlates significantly with muscle in the whole body (57). L3 is the typical location for evaluating muscle mass through CT, but not all patients have abdominal CT as a routine examination, and chest CT was used as a supplement (58, 59). Hamaguchi et al. (60) reported an apparent correlation between psoas muscle mass and total body skeletal muscle. Leone et al. (28) found that both L3-SMI and TMT could diagnose sarcopenia in PCNSL patients, while TMT seemed to be better as it showed a close relationship with grip strength (61).

Although CT is available for most clinical settings and could be used to obtain healthy massages, cut-offs to judge LMM are not yet well determined (13). EWGSOP2 has provided recommendations for cut-off points focusing on European populations and using normative references.

This review has several strengths. First, sarcopenia was assessed at the similar time point in all studies: all included articles assessed sarcopenia before any treatment. Second, we used a professional librarian’s overall search strategy to ensure that all related studies were included. Third, we also performed subgroup analyses by disease category when considering hematologic malignancies.

There are some limitations. First, sarcopenia was identified by LMM alone. As EWGSOP2 updated the definition of sarcopenia, the assessment should focus on muscle strength in future studies. Second, the diseases themselves are tough to grasp, such as the pathophysiology of Hodgkin’s lymphoma and non-Hodgkin’s is different, while the present study’s subgroup division is the result of much deliberation and trial. Third, in this study, most of the studies were concentrated on lymphoma, and the studies on other hematological malignancies were insufficient. Although sarcopenia had an adverse prognostic effect on AML, MM, and PCNSL in this study, this result is unreliable due to the few included studies and patients. Fourth, all analyses were retrospective and written in English, which may lead to selection bias. Fifth, most studies did not discuss the correlation between frailty and prognosis. As one of the most common geriatric syndromes, frailty could affect disease outcomes like OS.

Recently, Tan et al. (62) conducted a cohort study investigating sarcopenic predictive value in the prognosis of 49 treatment-naïve patients with T−cell lymphoblastic lymphoma. Since the study included 23 minors, it did not meet our inclusion criteria. In this research, sarcopenia was not associated with OS or PFS.

In the future, large-sample multicenter high-quality studies will be needed.

## Conclusion

We found a high prevalence of sarcopenia in hematologic malignancies patients, and the prognosis of patients with sarcopenia is worse, especially AML and DLBCL. As a result, we should take corresponding prevention and treatment measures to reduce the incidence of sarcopenia. There is a dilemma in treating patients with sarcopenia: toxicity versus efficacy. Clinicians should conduct a comprehensive assessment of these patients, including physical function status such as sarcopenia and frailty, to make individualized treatment decisions for patients with hematologic malignancies. Male patients with sarcopenia have worse disease outcomes, but this conclusion must be confirmed in future large-sample multicenter studies.

## Data availability statement

The original contributions presented in the study are included in the article/**Supplementary Material**. Further inquiries can be directed to the corresponding author.

## Author contributions

XFZ: Conceptualization, Data curation, Formal analysis, Methodology, Software, Writing – original draft, Writing – review & editing. LZ: Data curation, Formal analysis, Methodology, Software, Writing – original draft, Writing – review & editing. YZ: Data curation, Methodology, Resources, Writing – original draft. SJ: Conceptualization, Formal analysis, Methodology, Writing – original draft. TL: Formal analysis, Methodology, Software, Writing – original draft. XMZ: Data curation, Formal analysis, Software, Writing – original draft. XH: Conceptualization, Formal analysis, Funding acquisition, Methodology, Project administration, Resources, Supervision, Validation, Writing – original draft, Writing – review & editing.
