# Supplementary material for: Prevalence and prognostic value of baseline sarcopenia in hematologic malignancies: a systematic review
Source: Front Oncol. 2023 Dec 14;13:1308544. doi: 10.3389/fonc.2023.1308544 (PMC10755879; doi:10.3389/fonc.2023.1308544)
Supplement: Supplementary file 1 [file DataSheet_1.docx]

**Table S1: Search strategy by Embase, Medline, and Cochrane library via Ovid SP**

Database: EBM Reviews - Cochrane Central Register of Controlled Trials

--------------------------------------------------------------------------------

1 exp muscular atrophy/

2 (sarcopen* or myopen* or dynapon* or amyotroph* or myoatroph* or myophagis* or myodegenerat*).mp.

3 ((muscle or muscular) adj5 (atroph* or wast* or weak* or loss* or mass or degenerat*)).ti,ab.

4 1 or 2 or 3

5 exp Leukemia/

6 (leucocythaemi* or aleuk?emi* or leuc?emi* or leuk?emi*).mp.

7 exp Lymphoma/)

8 (lymphoma* or germinoblast* or reticulolymphosarcom* or ((lymhogranulom* or granulom* or germinoblastic or lymphocytic or lymphoid or lymph node) adj5 (sarcoma* or tumo?r or malignanc*))).mp.

9 exp Multiple Myeloma/

10 ((myelom* adj5 plasma cell) or myelom*).mp.

11 exp Myelodysplastic Syndromes/

12 (myeloplasti* or myelodysplas* or dysmyelopoietic* or mielodispl?sic*).mp.

13 exp Hematologic Neoplasms/

14 5 or 6 or 7 or 8 or 9 or 10 or 11 or 12 or 13

15 4 and 14

***************************

Database: Ovid MEDLINE(R) and Epub Ahead of Print, In-Process, In-Data-Review & Other Non-Indexed Citations, Daily and Versions

--------------------------------------------------------------------------------

1 exp muscular atrophy/

2 (sarcopen* or myopen* or dynapon* or amyotroph* or myoatroph* or myophagis* or myodegenerat*).mp.

3 ((muscle or muscular) adj5 (atroph* or wast* or weak* or loss* or mass or degenerat*)).ti,ab.

4 1 or 2 or 3

5 exp Leukemia/

6 (leucocythaemi* or aleuk?emi* or leuc?emi* or leuk?emi*).mp.

7 exp Lymphoma/

8 (lymphoma* or germinoblast* or reticulolymphosarcom* or ((lymhogranulom* or granulom* or germinoblastic or lymphocytic or lymphoid or lymph node) adj5 (sarcoma* or tumo?r or malignanc*))).mp.

9 exp Multiple Myeloma/

10 ((myelom* adj5 plasma cell) or myelom*).mp.

11 exp Myelodysplastic Syndromes/

12 (myeloplasti* or myelodysplas* or dysmyelopoietic* or mielodispl?sic*).mp.

13 exp Hematologic Neoplasms/

14 5 or 6 or 7 or 8 or 9 or 10 or 11 or 12 or 13

15 4 and 14

***************************

Database: Embase

--------------------------------------------------------------------------------

1 exp muscular atrophy/

2 (sarcopen* or myopen* or dynapon* or amyotroph* or myoatroph* or myophagis* or myodegenerat*).mp.

3 ((muscle or muscular) adj5 (atroph* or wast* or weak* or loss* or mass or degenerat*)).ti,ab.

4 1 or 2 or 3

5 exp hematologic malignancy/

6 (leucocythaemi* or aleuk?emi* or leuc?emi* or leuk?emi*).mp.

7 (lymphoma* or germinoblast* or reticulolymphosarcom* or ((lymhogranulom* or granulom* or germinoblastic or lymphocytic or lymphoid or lymph node) adj5 (sarcoma* or tumo?r or malignanc*))).mp.

8 ((myelom* adj5 plasma cell) or myelom*).mp.

9 exp Myelodysplastic Syndromes/

10 (myeloplasti* or myelodysplas* or dysmyelopoietic* or mielodispl?sic*).mp.

11 5 or 6 or 7 or 8 or 9 or 10

12 4 and 11

***************************

**Table S2: The reasons for the exclusion of full-text screening.**

| Study | Reason for the exclusion |
| --- | --- |
| [1] | This article applied the same dataset with an included study.  Exclusion study types: letter. |
| [2] | This article focuses on the muscle loss during immunochemotherapy, rather than baseline status. |
| [3] | Sarcopenia was evaluated before hematopoietic stem cell transport (HSCT), rather than baseline status. |
| [4] | Sarcopenia was evaluated before hematopoietic stem cell transport (HSCT), rather than baseline status. |
| [5] | Sarcopenia was evaluated before hematopoietic stem cell transport (HSCT), rather than baseline status. |
| [6] | Sarcopenia was evaluated before hematopoietic stem cell transport (HSCT), rather than baseline status. |
| [7] | Sarcopenia was evaluated before hematopoietic stem cell transport (HSCT), rather than baseline status. |
| [8] | Sarcopenia was evaluated before hematopoietic stem cell transport (HSCT), rather than baseline status. |
| [9] | Sarcopenia was evaluated before hematopoietic stem cell transport (HSCT), rather than baseline status. |
| [10] | Not use acceptable diagnostic criteria (cut-off value): least square root of sum of squared sensitivity plus squared 100-specificity. |
| [11] | Not use acceptable diagnostic criteria (cut-off value): Z-scores from a Caucasian population; Z-scores of 0 indicating the mean muscle mass and muscle density in the healthy population; equal to or below -1 was defined as LMM. |
| [12] | Not reported a total sarcopenia prognostic data in malignant haematology cancer patients. |
| [13] | Not use acceptable diagnostic criteria: sarcopenia diagnosed by low skeletal muscular density (SMD). |
| [14] | Not use acceptable diagnostic criteria: sarcopenia diagnosed by low skeletal muscular density (SMD). |
| [15] | This article applied the same dataset with an included study. |
| [16] | Sarcopenia was evaluated before hematopoietic stem cell transport (HSCT), rather than baseline status.  Exclusion study types: conference abstract. |
| [17] | Sarcopenia was evaluated before hematopoietic stem cell transport (HSCT), rather than baseline status.  Exclusion study types: conference abstract. |
| [18] | Sarcopenia was evaluated before hematopoietic stem cell transport (HSCT), rather than baseline status.  Exclusion study types: conference abstract. |
| [19] | Sarcopenia was evaluated before hematopoietic stem cell transport (HSCT), rather than baseline status.  Exclusion study types: conference abstract. |
| [20] | Sarcopenia was evaluated before hematopoietic stem cell transport (HSCT), rather than baseline status.  Exclusion study types: conference abstract.  Not prognostic data reported. |
| [21] | This article applied the same dataset with an included study.  Exclusion study types: conference abstract. |
| [22] | This article applied the same dataset with an included study.  Exclusion study types: conference abstract. |
| [23] | This article applied the same dataset with an included study.  Exclusion study types: conference abstract. |
| [24] | This article applied the same dataset with an included study.  Exclusion study types: conference abstract. |
| [25] | This article applied the same dataset with an included study.  Exclusion study types: conference abstract. |
| [26] | This article applied the same dataset with an included study.  Exclusion study types: conference abstract. |
| [27] | This article applied the same dataset with an included study.  Exclusion study types: conference abstract. |
| [28] | This article applied the same dataset with an included study.  Exclusion study types: conference abstract. |
| [29] | This article applied the same dataset with an included study.  Exclusion study types: conference abstract. |
| [30] | This article applied the same dataset with an included study.  Exclusion study types: conference abstract. |
| [31] | This article applied the same dataset with an included study.  Exclusion study types: conference abstract. |
| [32] | This article applied the same dataset with an included study.  Exclusion study types: conference abstract. |
| [33] | This article applied the same dataset with an included study.  Exclusion study types: conference abstract. |
| [34] | This article applied the same dataset with an included study.  Exclusion study types: conference abstract. |
| [35] | Exclusion study types: conference abstract. |
| [36] | Exclusion study types: conference abstract. |
| [37] | Exclusion study types: conference abstract. |
| [38] | Exclusion study types: conference abstract. |
| [39] | Exclusion study types: conference abstract. |

1. Lucijanic M, Korunic RH, Sedinic M, Kusec R, Pejsa V. Prognostic impact of psoas muscle index in patients with diffuse large B-cell lymphoma might be dependent on the immunochemotherapy type. *Leukemia and Lymphoma* 2021;**62(10)**:2535-2538.

2. Lucijanic M, Korunic RH, Sedinic M, Kusec R, Pejsa V. More pronounced muscle loss during immunochemotherapy is associated with worse clinical outcomes in newly diagnosed patients with diffuse large b-cell lymphoma with unfavorable features. *Therapeutics and Clinical Risk Management* 2021;**17**:1037-1044.

3. Nagayama T, Fujiwara SI, Kikuchi T, Onda K, Murahashi R, Nakashima H, et al. Impact of muscle mass loss assessed by computed tomography on the outcome of allogeneic stem cell transplantation. *LEUKEMIA & LYMPHOMA* 2022;**63**:1694-1700.

4. Williams A, Baruah D, Patel J, Szabo A, Chhabra S, Dhakal B, et al. Prevalence and significance of sarcopenia in multiple myeloma patients undergoing autologous hematopoietic cell transplantation. *Bone Marrow Transplant* 2021;**56**:225-231.

5. Lin RJ, Michaud L, Lobaugh SM, Nakajima R, Mauguen A, Elko TA, et al. The geriatric syndrome of sarcopenia impacts allogeneic hematopoietic cell transplantation outcomes in older lymphoma patients. *LEUKEMIA & LYMPHOMA* 2020;**61**:1833-1841.

6. Armenian SH, Iukuridze A, Teh JB, Mascarenhas K, Herrera A, McCune JS, et al. Abnormal body composition is a predictor of adverse outcomes after autologous haematopoietic cell transplantation. *Journal of Cachexia, Sarcopenia and Muscle* 2020;**11**:962-972.

7. Ando T, Fujisawa S, Teshigawara H, Matsumura A, Sakuma T, Suzuki T, et al. Computed tomography-defined sarcopenia: prognostic predictor of nonrelapse mortality after allogeneic hematopoietic stem cell transplantation: a multicenter retrospective study. *INTERNATIONAL JOURNAL OF HEMATOLOGY* 2020;**112**:46-56.

8. Armenian SH, Xiao M, Berano Teh J, Lee B, Chang HA, Mascarenhas K, et al. Impact of Sarcopenia on Adverse Outcomes After Allogeneic Hematopoietic Cell Transplantation. *Journal of the National Cancer Institute* 2019;**111**:837-844.

9. Caram MV, Bellile EL, Englesbe MJ, Terjimanian M, Wang SC, Griggs JJ, et al. Sarcopenia is associated with autologous transplant-related outcomes in patients with lymphoma. *LEUKEMIA & LYMPHOMA* 2015;**56**:2855-2862.

10. Guo J, Cai P, Li P, Cao C, Zhou J, Dong L, et al. Body Composition as a Predictor of Toxicity and Prognosis in Patients with Diffuse Large B-Cell Lymphoma Receiving R-CHOP Immunochemotherapy. *Current Oncology* 2021;**28**:1325-1337.

11. Rier HN, Kharagjitsing H, van Rosmalen J, van Vugt JLA, Westerweel PE, de Jongh E, et al. Prognostic impact of low muscle mass and low muscle density in patients with diffuse large B-cell lymphoma. *Leukemia and Lymphoma* 2020;**61(7)**:1618-1626.

12. Go SI, Park MJ, Song HN, Kim HG, Kang MH, Kang JH, et al. A comparison of pectoralis versus lumbar skeletal muscle indices for defining sarcopenia in diffuse large B-cell lymphoma - two are better than one. *Oncotarget* 2017;**8**:47007-47019.

13. Chu MP, Lieffers J, Ghosh S, Belch A, Chua NS, Fontaine A, et al. Skeletal muscle density is an independent predictor of diffuse large B-cell lymphoma outcomes treated with rituximab-based chemoimmunotherapy. *Journal of Cachexia, Sarcopenia and Muscle* 2017;**8**:298-304.

14. Chu MP, Lieffers J, Ghosh S, Belch AR, Chua NS, Fontaine A, et al. Skeletal muscle radio-density is an independent predictor of response and outcomes in follicular lymphoma treated with chemoimmunotherapy. *PLoS One* 2015;**10(6) (no pagination)**

15. Camus V, Lanic H, Kraut J, Modzelewski R, Clatot F, Picquenot JM, et al. Prognostic impact of fat tissue loss and cachexia assessed by computed tomography scan in elderly patients with diffuse large B-cell lymphoma treated with immunochemotherapy. *EUROPEAN JOURNAL OF HAEMATOLOGY* 2014;**93**:9-18.

16. Shibasaki Y, Kobayashi K, Suwabe T, Fuse K, Narita M, Sone H, et al. Depletion of Pre-Transplant Skeletal Muscle Is a Significant Poor Prognostic Factor in Allogeneic Hematopoietic Cell Transplantation. *BLOOD* 2019;**134(Supplement 1)**:3322.

17. Lin RJ, Michaud L, Lobaugh SM, Nakajima R, Elko TA, Ruiz JD, et al. The Geriatric Syndrome of Sarcopenia Impacts Allogeneic Hematopoietic Cell Transplantation Outcomes in Combination with Multi-Morbidity and Functional Impairment. *BLOOD* 2019;**134(Supplement 1)**:4508.

18. Armenian SH, Yang D, Teh JB, Lee B, Chang H, Kristen M, et al. Sarcopenia is a clinically relevant and independent predictor of adverse outcomes after allogeneic hematopoietic cell transplantation. *Blood Conference: 59th Annual Meeting of the American Society of Hematology, ASH* 2017;**130**

19. Ando T, Fujisawa S, Sakuma T, Teshigawara H, Matsumura A, Suzuki T, et al. Value of computed tomography-defined sarcopenia: A prognostic predictor of non-relapse mortality following allogeneic hematopoietic stem cell transplantation in patients with acute myeloid leukemia and myelodysplastic syndrome. *Blood Conference: 59th Annual Meeting of the American Society of Hematology, ASH* 2017;**130**

20. Neto AC, Del Guerra Carvalho Moraes B, Rocha IMG, Bezerra FA, Medeiros GOC, Alves LBO, et al. Association of sarcopenia with toxicities and survival after autologous hematopoietic stem cell transplantation for adults with lymphomas. *Blood Conference: 60th Annual Meeting of the American Society of Hematology, ASH* 2018;**132**

21. Nakamura N, Shibata Y, Matsumoto T, Nakamura H, Kitagawa J, Ninomiya S, et al. Sarcopenia and adipopenia are independent prognostic factors in patients with acute myeloid leukemia. *BLOOD* 2015;**126(23)**:4953.

22. Zilioli VR, Albano D, Arcari A, Merli F, Coppola A, Besutti G, et al. Sarcopeniaisanindependentprognosticfactor in elderly male patients with classical hodgkin lymphoma: Results from a multicenter experience. *HEMATOLOGICAL ONCOLOGY* 2021;**39(SUPPL 2)**:285-286.

23. Lucijanic M, Korunic RH, Sedinic M, Kusec R, Pejsa V. ABCL-018: Muscle Loss During Immunochemotherapy for Diffuse Large B-Cell Lymphoma and its Clinical and Prognostic Associations. *Clinical Lymphoma, Myeloma and Leukemia* 2021;**21(Supplement 1)**:S376.

24. Leone R, Sferruzza G, Calimeri T, Steffanoni S, Conte G, De Cobelli F, et al. Quantitative muscle mass biomarkers are independent prognosis factors in primary central nervous system lymphoma: The role of l3-skeletal muscle index and temporal muscle thickness. *NEURO-ONCOLOGY* 2021;**23(SUPPL 2)**:ii48.

25. Rier HN, Kharagjitsing H, Van Rosmalen J, Van Vugt J, Westerweel PE, De Jongh E, et al. Prognostic Impact of Low Muscle Mass and Muscle Density in Patients with Diffuse Large B-Cell Lymphoma. *Journal of Geriatric Oncology* 2019;**10(6 Supplement 1)**:S38.

26. Nakamura N, Ninomiya S, Nakamura H, Kitagawa J, Hara T, Saito K, et al. Prognostic impact of skeletal muscle assessed by computed tomography scan in patients with acute myeloid leukemia. *HemaSphere* 2018;**2(Supplement 2)**:78.

27. Go SI, Park MJ, Lee GW. Clinical relevance of sarcopenia in diffuse large B-cell lymphoma-two are better than one. *HAEMATOLOGICA* 2017;**102(Supplement 2)**:398.

28. Go SI, Park MJ, Kim HG, Kang MH, Lee GW. Prognostic significance of sarcopenia in patients with diffuse large B-cell lymphoma treated with rituximab plus CHOP (R-CHOP). *HAEMATOLOGICA* 2016;**101(Supplement 1)**:686-687.

29. Nakamura N, Matsumoto T, Shibata Y, Kitagawa J, Kanemura N, Hara T, et al. Sarcopenia and adipopenia are independent prognostic factors in patients with acute myeloid leukemia. *ANNALS OF ONCOLOGY* 2015;**7)**:vii84.

30. Lanic H, Kraut J, Modzelewski R, Clatot F, Picquenot JM, Contentin N, et al. Clinical relevance of cachexia assessed by an anthropometric tool in elderly patients with diffuse large b-cell lymphoma treated by immunochemotherapy. *Blood Conference: 55th Annual Meeting of the American Society of Hematology, ASH* 2013;**122**

31. Chu MP, Lieffers J, Belch AR, Chua N, Fontaine A, Sangha R, et al. Indolent non-hodgkin lymphoma: Skeletal muscle density predicts overall survival with rituximab based chemotherapy. *Blood Conference: 55th Annual Meeting of the American Society of Hematology, ASH* 2013;**122**

32. Chu MP, Lieffers J, Belch AR, Chua N, Fontaine A, Sangha R, et al. Aggressive non-hodgkin lymphoma: Predictive value of sarcopenia and skeletal muscle density on prognosis with rituximab containing therapy. *Blood Conference: 55th Annual Meeting of the American Society of Hematology, ASH* 2013;**122**

33. Lanic H, Kraut J, Modzelewski R, Picquenot JM, Loschi M, Stamatoullas A, et al. Sarcopenia determined by computed tomography imaging is an independant prognostic factor in elderly patients with diffuse large B CELL lymphoma treated by immunochemotherapy. *Blood Conference: 54th Annual Meeting of the American Society of Hematology, ASH* 2012;**120**

34. Kraut J, Modzelewski R, Lanic H, Loschi M, Alcantara M, Picquenot JM, et al. Sarcopenia determined by computed tomography (CT) imaging is a better prognosis factor than albuminemia or Charlson index in elderly patients with diffuse large B-cell lymphoma (DLBCL). *Blood Conference: 53rd Annual Meeting of the American Society of Hematology, ASH* 2011;**118**

35. Menghini A, Bonm A, Graber J. Sarcopenia Measured by Temporalis Muscle Thickness Independently Predicts Early Relapse and Short Survival in Primary CNS Lymphoma. *Neurology Conference: 74th Annual Meeting of the American Academy of Neurology, AAN* 2022;**98**

36. Drory SB, Forget MF, Ting Wang H, Fleury I, Ahmad I, Marchand L. Sarcopenia and clinical evaluation in predicting treatment tolerability and outcomes in aggressive non- Hodgkin B cell lymphoma. *Journal of Clinical Oncology Conference* 2020;**38**

37. Teranaka H, Fujisawa S, Sakuma T, Teshigawara H, Matsumura A, Ando T, et al. Clinical impact of sarcopenia and skeletal muscle mass change during chemotherapy on outcomes of diffuse large b-cell lymphoma. *Blood Conference: 59th Annual Meeting of the American Society of Hematology, ASH* 2017;**130**

38. Vaxman I, Schlesinger A, Goldberg N, Lahav M, Gafter A, Lando S, et al. Muscle mass in elderly patients with DLBCL treated with RCHOP. *Blood Conference: 58th Annual Meeting of the American Society of Hematology, ASH* 2016;**128**

39. Bonm A, Menghini A, Graber J. Sarcopenia as measured by temporalis muscle width is a predictor of survival in primary CNS lymphoma. *NEURO-ONCOLOGY* 2021;**23(SUPPL 6)**:vi145.

**Table S3: Risk of bias of the included studies using Newcastle-Ottawa Scale**

|  | **Selection (1)** | | | | **Comparability (2)** | **Outcome (3)** | | |
| --- | --- | --- | --- | --- | --- | --- | --- | --- |
|  | **Representativeness of the exposed cohort** | **Selection of the non-exposed cohort** | **Ascertainment of exposure** | **Outcomes were not present at study initiation** | **Comparability of cohorts on the basis of the design or analysis** | **Assessment of outcome** | **Was follow-up long enough for outcome to occur** | **Adequacy of follow-up** |
| Nandakumar, 2023 | 1 | 1 | 1 | 1 | 2 | 1 | 1 | 0 |
| Ferraro, 2022 | 1 | 1 | 1 | 1 | 0^&^ | 1 | 1 | 0 |
| Albano, 2022 | 1 | 1 | 1 | 1 | 2 | 1 | 1 | 1* |
| Sun, 2022 | 1 | 1 | 1 | 1 | 2 | 1 | 1 | 0 |
| Lucijanić, 2021^$^ | 1 | 1 | 1 | 1 | 0^&^ | 0 | 1 | 0 |
| Albano, 2022 | 1 | 1 | 1 | 1 | 2 | 1 | 1 | 1* |
| Zilioli,2021 | 1 | 1 | 1 | 1 | 2 | 1 | 1 | 1* |
| Leone,2021 | 1 | 1 | 1 | 1 | 2 | 1 | 1 | 1* |
| Koyuncu,2021 | 1 | 1 | 1 | 1 | 2 | 1 | 1 | 0 |
| Jung, 2021 | 1 | 1 | 1 | 1 | 2 | 1 | 1 | 0 |
| Jullien, 2021 | 1 | 1 | 1 | 1 | 1^#^ | 1 | 1 | 1* |
| Furtner,2021 | 1 | 1 | 1 | 1 | 0^&^ | 1 | 1 | 0 |
| Besutti, 2021 | 1 | 1 | 1 | 1 | 0^&^ | 1 | 1 | 1* |
| Iltar, 2020 | 1 | 1 | 1 | 1 | 2 | 1 | 1 | 0 |
| Nakamura, 2019 | 1 | 1 | 1 | 1 | 2 | 1 | 1 | 0 |
| Burkart, 2019 | 1 | 1 | 1 | 1 | 2 | 1 | 1 | 0 |
| Xiao, 2016 | 1 | 1 | 1 | 1 | 2 | 1 | 1 | 0 |
| Takeoka, 2016 | 1 | 1 | 1 | 1 | 0^&^ | 1 | 1 | 0 |
| Go, 2016 | 1 | 1 | 1 | 1 | 2 | 1 | 1 | 1* |
| Nakamura, 2015 | 1 | 1 | 1 | 1 | 2 | 1 | 1 | 0 |
| Lanic, 2014 | 1 | 1 | 1 | 1 | 2 | 1 | 1 | 0 |

* Provide follow-up details. ^#^ No comparison of chemotherapies between sarcopenic and nonsarcopenic group. ^&^ Not report the comparability of cohorts on the baseline. ^$^ This article is a short report.

**Table S4 Impact of sarcopenia on survival outcomes**

| Authors, year | Mean/median follow-up (range) | OS | | PFS | |
| --- | --- | --- | --- | --- | --- |
|  |  | univariate HR, 95% CI | multivariate HR, 95% CI | univariate HR, 95% CI | multivariate HR, 95% CI |
| Nandakumar, 2023 | 72 months (95% CI, 63–83) | - | - | - | - |
| Ferraro, 2022 | at least two years or until death | 0.61, 0.31-1.21, p = 0.16 | - | 0.65, 0.33-1.27, p = 0.20 | - |
| Albano, 2022 | 47.5 months (6–147 months) | 3.275, 1.592–6.737, p=0.001 | 3.661, 1.111–7.321, p=0.001 | 3.725, 1.934–7.174, p<0.001 | 3.881, 1.009–7.021, p=0.001 |
| Sun, 2022 | 30.7 months | 2.055, 1.254–3.367, p = 0.004 | 1.887, 1.071–3.324, p = 0.028 | - | - |
| Albano, 2022 | 50 months | 1.574, 0.736–3.365, p = 0.262 | - | 0.125, 0.062–0.253, p < 0.001 | 0.031, 0.007–0.132, p < 0.001 |
| Lucijanić, 2021 | 51 months | - | - | 4.91, p = 0.009 | 5.09, p = 0.042 |
| Zilioli,2021 | 5.9 years (4.7–6.6 years) | male: 2.29, 1.03–5.10, p = 0.042 | male: 2.69, 0.97–7.46, p = 0.057 | male: 2.43, 1.27–4.66, p = 0.008 | male: 3.09, 1.43–6.64, p = 0.004 |
| Leone,2021 | 23 months (12–40 months) | 4.61, 1.73–12.29, p = 0.002) | 4.94, 1.78–13.68, p = 0.002 | 4.57, 1.87–11.12, p = 0.001 | 4.39, 1.68–11.49, p < 0.001 |
| Koyuncu,2021 | 21.7 months (1.51-74.97 months) | 2.43, 1.02-5.77, p = 0.028 | - | - | - |
| Jung, 2021 | 21.5 months (1–69 months) | - | 2.64, 1.38–5.07, p = 0.003 | - | 1.73, 0.98–3.07, p = 0.060 |
| Jullien, 2021 | 36.6 months (29.9–45.1 months) | 1.20, 0.74-1.93, p = 0.46 | - | 1.27, 0.90-1.80, p = 0.18 | - |
| Furtner,2021 | NA | 3.189, 2.097–4.848, p < 0.001 | 2.504, 1.608–3.911, p < 0.001 | - | - |
| Besutti, 2021 | 30 months (24–34 months) | 0.70, 0.26–1.85, p = 0.469 | 0.67, 0.25–1.82; p = 0.431 | 1.47, 0.77–2.82, p = 0.248 | 1.53, 0.78–3.00, p = 0.216 |
| Iltar, 2020 | NA | 2.77, 1.344–5.711, p = 0.006 | 2.554, 1.117-5.838, p = 0.026 | 1.834, 0.975–3.453, p = 0.060 | 1.560, 0.752-3.236, p = 0.232 |
| Nakamura, 2019 | 13.8 months (0.5–146.5 months) | - | 2.27, 1.11–4.79, p < 0.05 | - | 9.39, 3.19–30.62, p < 0.001 |
| Burkart, 2019 | 33 months | 4.8, p = 0.004 | 4.19, 0.42–41.35, p = 0.22 | - | - |
| Xiao, 2016 | NA | - | - | - | - |
| Takeoka, 2016 | 2.3 years | 0.51, 0.2-1.32, p = 0.16 | - | - | - |
| Go, 2016 | 60.9 months (0.2–145.3 months) | 2.681, 1.674–4.294, p < 0.001 | 2.112, 1.299-3.435, p = 0.003 | 2.583, 1.643–4.059, p < 0.001 | 2.074, 1.298-3.312, p = 0.002 |
| Nakamura, 2015 | 50.4 months (3.0–121.9 months) | - | 1.68, 0.85–3.51, p = 0.1379 | - | 1.64, 0.89–3.12, p = 0.1137 |
| Lanic, 2014 | 39 months | 3.22, 1.73-5.98, p < 0.001 | 2.07, 1.01-4.26, p = 0.04 | 2.66, 1.5-4.7, p < 0.001 | 2.24, 1.21 – 4.12, p =0.01 |

**Figure S1 Meta-regression of the effect of age on prevalence of sarcopenia**


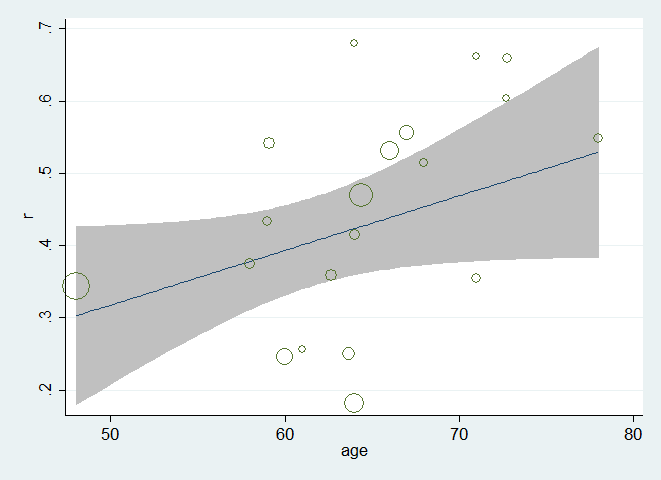


**Figure S2 Meta-regression of the effect of study regions on prevalence of sarcopenia.**

**Figure S3 The prevalence of sarcopenia: elder v.s. non-elder**


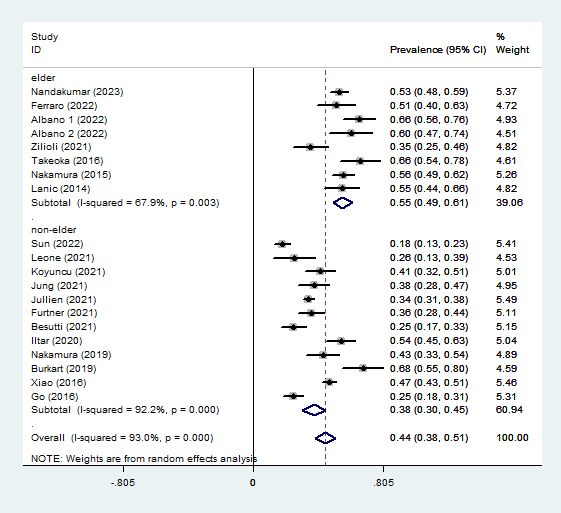


**Figure S4 The prevalence of sarcopenia: different ways to diagnose sarcopenia**


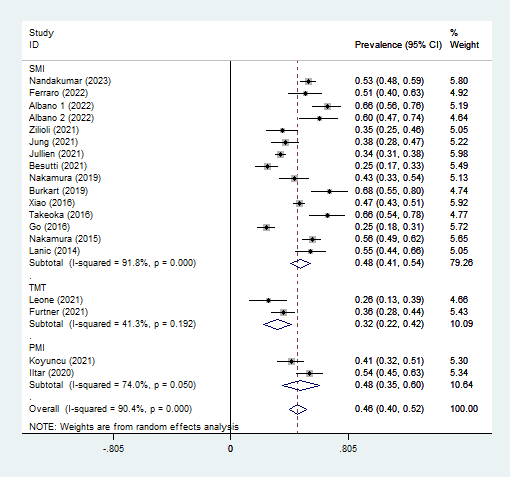


**Figure S5 different types of disease**


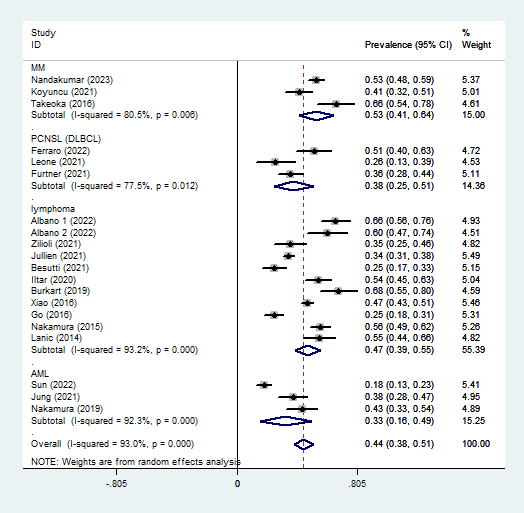


**Figure S6 excluding Ferraro et al. study, the heterogeneity of pooled OS decreased significantly**


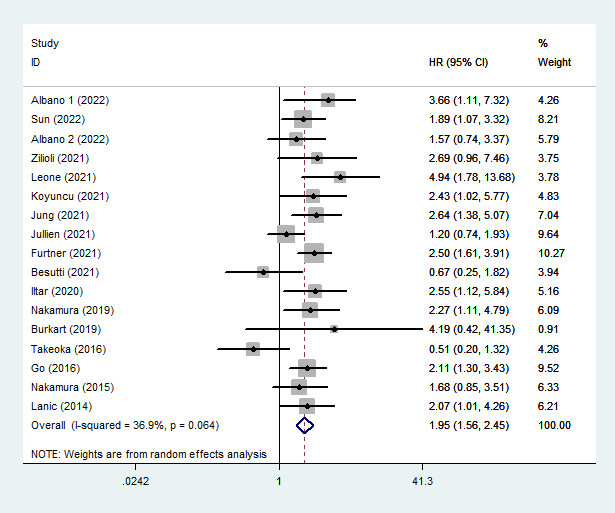


**Figure S7 When we excluded three of the articles [26, 36, 46], there was an apparent decrease in the heterogeneity of pooled PFS**


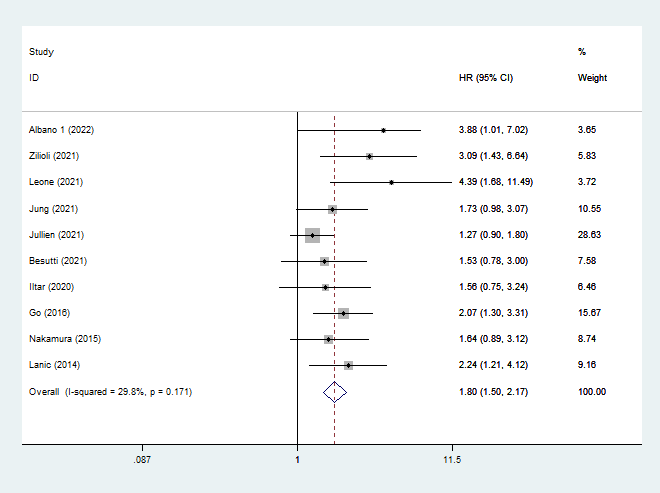


**Figure S8 Meta-regression of the effect of age on OS (a) and PFS (b)**


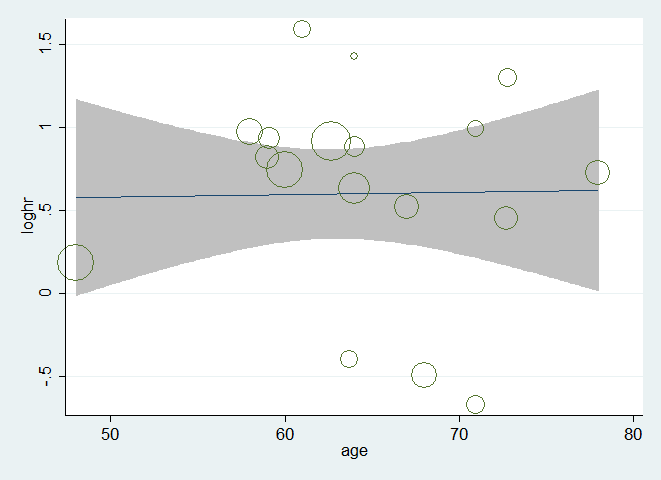
(a)


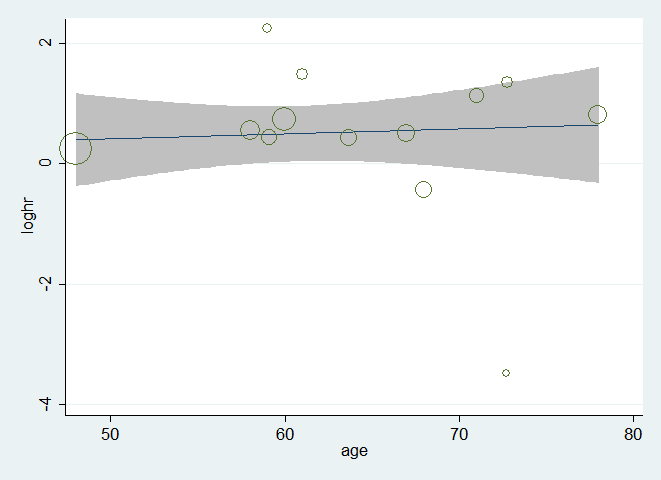


(b)

**Figure S9 There was no evidence of publication bias in the papers that described the prevalence of sarcopenia [19 studies; Begg's test (a): P = 0.230; Egger's test (b): P = 0.104]**


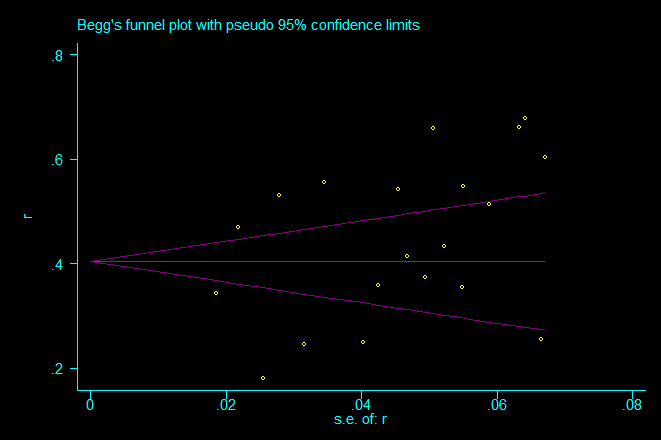


(a)


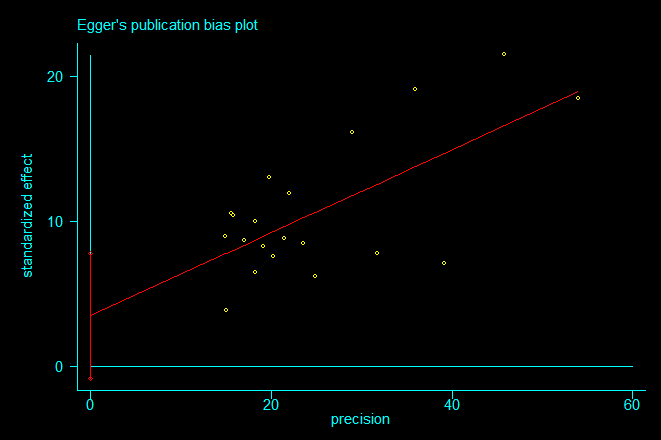


(b)

**Figure S10** **There was no evidence of publication bias in the papers that described the prognostic value [OS: 18 studies; Begg's test (a): P = 0.544; Egger's test (b): P = 0.848; PFS: 13 studies; Begg's test (c): P = 0.428; Egger's test (d): P = 0.862]**


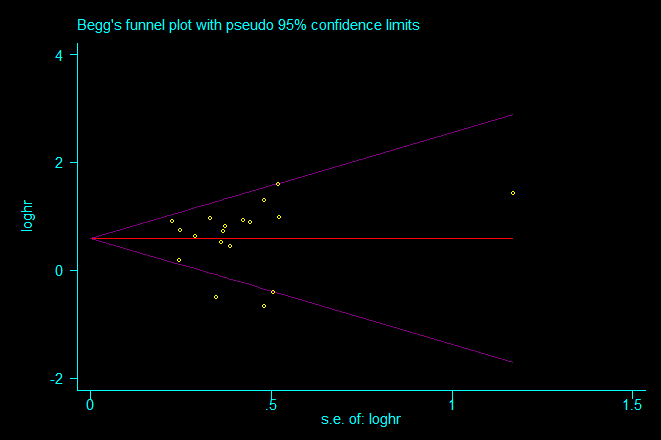


(a)


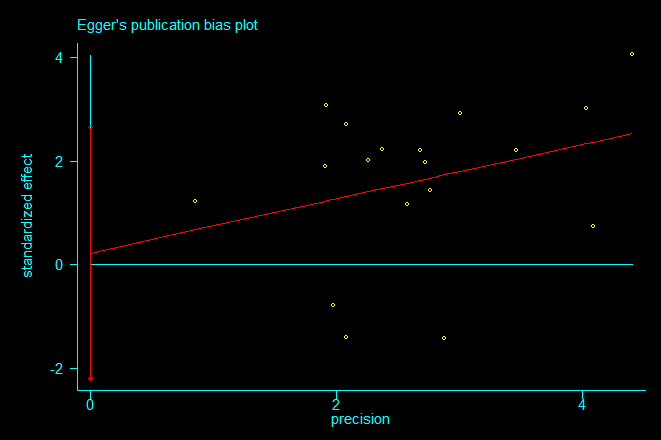


(b)


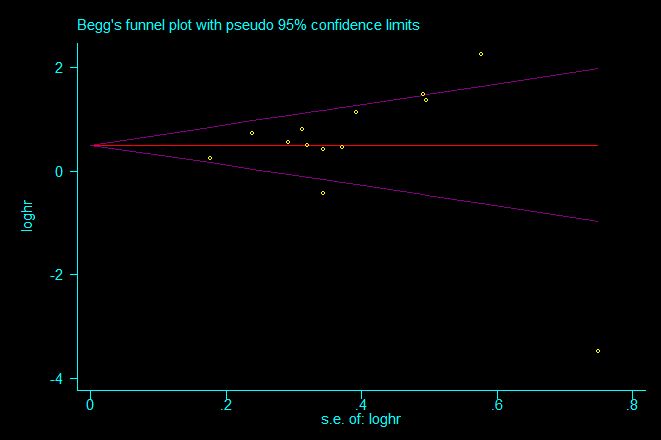


(c)


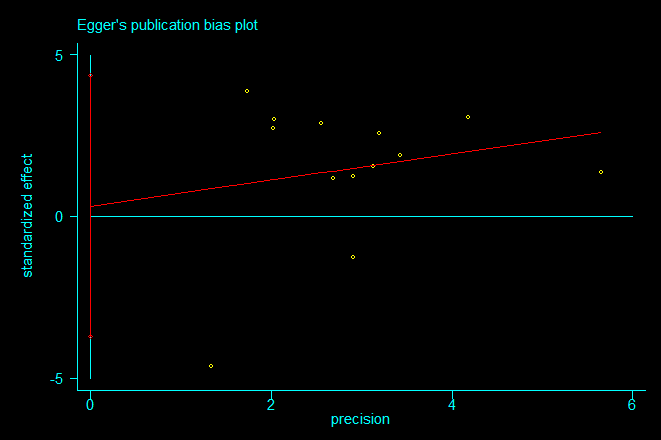


(d)

**Figure S11 Sensitivity analysis detected that any individual study did not significantly affect the pooled prevalence of sarcopenia (a) or pooled prognostic value (OS, PFS; b-c)**

**
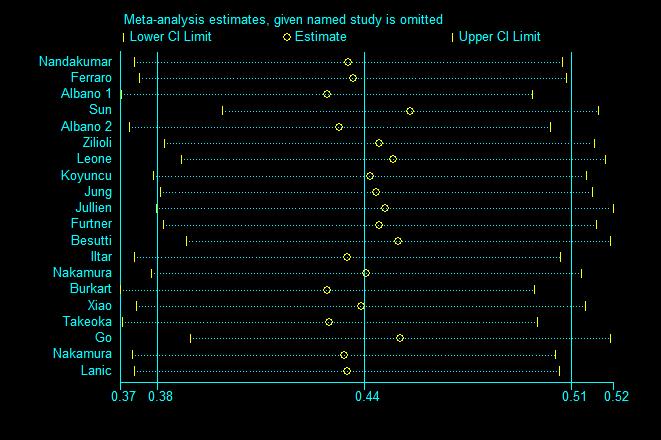
**(a)

**
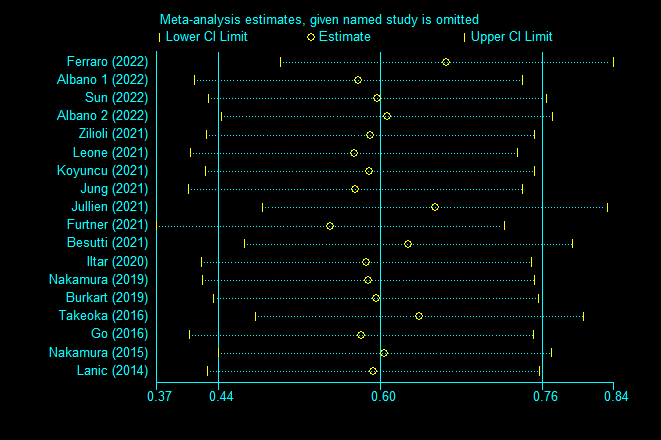
**(b)


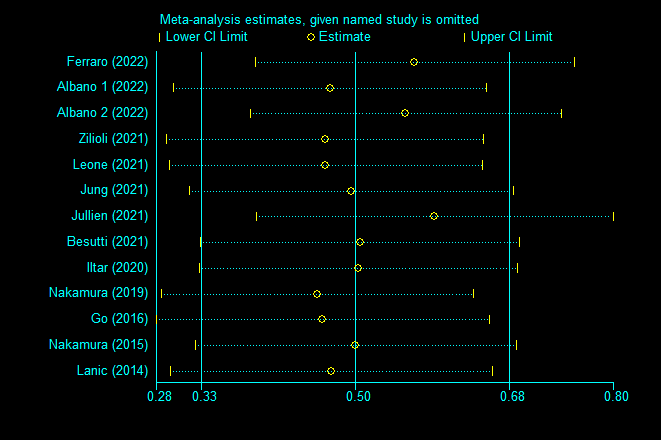


(c)
